# Strain-induced enhancement of plasma dispersion effect and free-carrier absorption in SiGe optical modulators

**DOI:** 10.1038/srep04683

**Published:** 2014-04-15

**Authors:** Younghyun Kim, Mitsuru Takenaka, Takenori Osada, Masahiko Hata, Shinichi Takagi

**Affiliations:** 1 Department of Electrical Engineering and Information Systems, The University of Tokyo, 7-3-1 Hongo, Bunkyo-ku, Tokyo 113-8656, Japan; 2 Sumitomo Chemical Co., Ltd., 6 Kitahara, Tsukuba, Ibaraki 300-3294, Japan

## Abstract

The plasma dispersion effect and free-carrier absorption are widely used to change refractive index and absorption coefficient in Si-based optical modulators. However, the weak free-carrier effects in Si cause low modulation efficiency, resulting in large device footprint and power consumption. Here, we theoretically and experimentally investigate the enhancement of the free-carrier effects by strain-induced mass modulation in silicon-germanium (SiGe). The application of compressive strain to SiGe reduces the conductivity effective mass of holes, resulting in the enhanced free-carrier effects. Thus, the strained SiGe-based optical modulator exhibits more than twice modulation efficiency as large as that of the Si modulator. To the best of our knowledge, this is the first demonstration of the enhanced free-carrier effects in strained SiGe at the near-infrared telecommunication wavelength. The strain-induced enhancement technology for the free-carrier effects is expected to boost modulation efficiency of the most Si-based optical modulators thanks to high complementary metal-oxide-semiconductor (CMOS) compatibility.

The plasma dispersion effect and free-carrier absorption are well known physical phenomena to change optical constants: refractive index (*n*) and absorption coefficient (*α*) in semiconductors. In particular, these effects are the most promising for silicon (Si) to build-up optical modulators as previously shown in the study by Soref and Bennett^1^. Thus, Si optical modulators based on the free-carrier effects have been demonstrated thus far by carrier modulation through injection, depletion and accumulation^2^,^3^,^4^,^5^,^6^,^7^,^8^,^9^,^10^,^11^,^12^,^13^. In particular, a depletion-based Mach-Zehnder interferometer (MZI) optical modulator, in which the refractive index of the phase shifters are modulated by carrier depletion, is one of the most promising modulators in terms of modulation speed and optical bandwidth. However, the weak plasma dispersion and free-carrier absorption in Si cause the low modulation efficiency, resulting in a long device length for MZI modulators.

To supplement the weak plasma dispersion effect and free-carrier absorption in Si, the introduction of ring resonators instead of MZIs^2^,^11^, and slow-light structures using photonic crystals^12^,^13^ have been investigated, while the optical bandwidth has also been reduced in such structures. Hence, the enhancement of the plasma dispersion effect and free-carrier absorption is indispensable for Si-based optical modulators.

According to the Drude model, the plasma dispersion effect and free-carrier absorption are expressed by 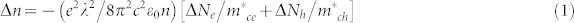

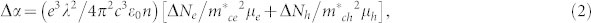
where *e* is the elementary charge, *ε*_0_ is the permittivity in vacuum, *c* is the speed of light in vacuum, *λ* is the wavelength, *n* is the unperturbed refractive index, m*_ce_ and m*_ch_ are the conductivity effective masses of electrons and holes, and *μ_e_* and *μ_h_* are the mobilities of electrons and holes, respectively. The Drude model describes the changes in the refractive index and absorption coefficient arising from a change in the plasma frequency of free carriers, which is dependent on not only the number of free carriers but also their conductivity effective masses. According to the model, the change in the refractive index induced by the plasma dispersion effect is inversely proportional to the conductivity effective masses of electrons and holes^14^. Therefore, the lighter the conductivity masses become, the greater the plasma dispersion is. In complementary metal-oxide-semiconductor (CMOS) technology, the application of strain to Si has been widely used to achieve lighter conductivity masses and higher mobilities in the channels of transistors, which can overcome the fundamental difficulty in scaling MOS transistors. Tensile strain and compressive strain are applied to Si n-channel MOS transistors and p-channel MOS transistors, respectively^15^,^16^,^17^. It was also experimentally observed that the plasma dispersion effect and free-carrier absorption in Si in the far-infrared wavelength range from 5 to 20 μm were modified by uniaxial strain mechanically applied to Si through strain-induced mass modulation^18^,^19^,^20^. Here, we report the enhancement of the plasma dispersion effect and free-carrier absorption by strain-induced mass modulation in a Si/strained SiGe/Si double-heterostructure waveguide with a lateral *pin* junction for carrier injection in the near-infrared wavelength range from 1.3 to 1.6 μm, which is the most important range of wavelengths for optical fiber communication.

The strain in SiGe coherently grown on Si is controllable via the Ge mole fraction of SiGe because the lattice constant of Ge is 4% larger than that of Si. Hence, a biaxial compressive strained SiGe film can be obtained when the thickness of SiGe grown on Si is less than its critical thickness^21^,^22^. The band structure of SiGe is highly affected by strain, resulting in the mass modulation of electrons and holes. Figure 1 shows the valance band structures of a light hole (LH) and heavy hole (HH) as a function of the wave vector in the *k_x_* [100]- *k_y_* [010] plane calculated by the *k·p* method when SiGe films with Ge fractions, *x* of 0, 0.15, and 0.3 are coherently grown on Si (001)^23^,^24^,^25^. In the case of Si (*x* = 0), the HH and LH bands are degenerate at the Γ point. The compressive strain splits this degeneracy; thus, the HH band is shifted to above the LH band in the case of strained Si_1−*x*_Ge*_x_* on Si (*x* > 0). The energy surfaces of the LH and HH bands become sharp at the minimum-energy Γ point, indicating that the biaxial compressive strain reduces the effective hole mass at the Γ point. Thus, the conductivity effective mass of holes also decreases, resulting in the enhanced free-carrier effects (Supplementary Fig. S2).

We numerically analyzed the modulation characteristics of a carrier-injection waveguide optical modulator with a biaxial compressive strained SiGe well in the heterostructure by performing a technology computer aided design (TCAD) simulation in conjunction with finite-difference optical mode analysis. Figure 2a shows a schematic of the device structure with a lateral *pin* junction for carrier injection. The solid lines in Fig. 2b show the optical attenuation characteristics of the SiGe optical modulator with a Ge fraction from 0 to 0.3 as a function of injected current density. Owing to the enhanced free-carrier absorption and the carrier confinement^4^ by the band offset^26^ in the SiGe layer (Supplementary Fig. S4), the optical attenuation increases with the Ge fraction. Figure 2c shows the change in the refractive index which has same trend as the attenuation characteristics. To identify the effect of carrier confinement on the modulation efficiency, the modulation characteristics considering only carrier confinement, in which the enhancement of the plasma dispersion effect and free-carrier absorption in SiGe are neglected, are calculated as shown by the broken lines in Figs. 2b and 2c. Although the carrier confinement partly contributes to enhancing the device performance, the strain effect has the most important role in enhancing the modulation characteristics. Figure 2d shows the current density required for 20 dB attenuation and the enhancement factor of optical attenuation against Si as a function of the Ge fraction. The current density required for 20 dB attenuation significantly decreases from 57 mA for Si (*x* = 0) to 15 mA for Si_0.7_Ge_0.3_. Hence, the modulation efficiency of the Si_0.7_Ge_0.3_-based in-line intensity modulator based on free-carrier absorption is predicted to be approximately 3.7 times as large as that of the Si-based modulator.

A *pin* injection-type optical modulator was fabricated by the conventional Si CMOS process. Figure 3a shows a cross-sectional transmission electron microscopy (TEM) image of the Si/SiGe/Si rib waveguide with a 600-nm-wide mesa. The Si/SiGe/Si heterostructure can be clearly observed in Fig. 3b. A Si-based device without a SiGe well was also fabricated as a control device by the same process.

Figure 3c shows the measured optical attenuation properties of the SiGe and Si in-line intensity modulators at the wavelength of 1.55 μm. The simulated optical attenuation properties, in which the changes in the Ge fraction and strain after device fabrication are taken into account (Supplementary Fig. S5), are also plotted as solid lines. As shown in Fig. 3c, the optical attenuation is increased by current injection through the *pin* junction owing to free-carrier absorption; the SiGe modulator exhibits higher attenuation than the Si modulator at the same current density. The experimental results of the SiGe device show fairly good agreement with the simulation results. Therefore, it is clearly shown that strain-induced mass modulation enhances the free-carrier absorption and improves the device performance. The injected current densities required for 20 dB attenuation are approximately 24 and 55 mA/mm for the SiGe and Si modulators, respectively. Thus, the modulation efficiency of the SiGe device is more than twice as large as that of the Si control device. Furthermore, the bias voltages are approximately 1.8 and 2.45 V for the SiGe and Si modulators, respectively. Therefore, the power consumption required for 20-dB attenuation is also reduced from 135 mW to 43 mW by introducing strained SiGe instead of Si.

The wavelength dependence of the optical attenuation of the strained SiGe and Si modulators is analyzed with varied currents in the wavelength range from 1.34 to 1.64 μm. Since the free-carrier effects are proportional to the square of the wavelength according to the Drude model, we plot the optical attenuation measured at injection currents of 20, 30, and 40 mA/mm as a function of the square of the wavelength in Fig. 4a. Linear relationships between the optical attenuation and the square of the wavelength are clearly observed, meaning that the optical attenuation arises from free-carrier absorption. From the wavelength dependences of the optical attenuation characteristics, we calculated the change in the effective refractive index using the Kramers-Kronig relations^1^. Since the measurable wavelength range is limited to from 1.34 to 1.64 μm, corresponding to the photon energy range from 0.756 to 0.925 eV, the free-carrier absorption spectra are extrapolated into the far-infrared wavelength range for the calculation^27^. Figure 4b shows the calculated changes in the effective refractive index at injection currents of 20, 30, and 40 mA/mm as a function of the square of the wavelength, which exhibit a linear relationship.

The changes in the optical constants of the SiGe layer are deduced from the wavelength dependences in Fig. 4 by taking into account the optical confinement factor, the strain relaxation during the dopant activation process, and the carrier concentration in the 50-nm-thick SiGe layer through the TCAD simulation. The changes in the optical constants of Si are also deduced in the same way. Figure 5 shows the changes in the refractive index and absorption coefficient change of 85%-strained Si_0.86_Ge_0.14_ and Si as functions of carrier concentration, in which the solid lines show the theoretical values calculated using the Drude model. The fairly good agreement between the experiment results and the theory clearly indicates that the plasma dispersion effect and free-carrier absorption are enhanced by strain-induced mass modulation in strained SiGe. Thus, the enhancement factors of 1.3 for Δ*n* and 1.7 for Δ*α* in 85%-strained Si_0.86_Ge_0.14_ have been successfully demonstrated.

In conclusion, we have demonstrated that the strain-induced enhancement of the plasma dispersion effect and free-carrier absorption in biaxial compressive strained SiGe is effective for boosting the modulation efficiency of Si-based optical modulators. The optical attenuation of the SiGe-based in-line intensity optical modulator is more than twice as large as that of the Si modulator. This is the first demonstration of enhanced free-carrier absorption in SiGe through strain-induced mass modulation for the telecommunication wavelength range from 1.3 to 1.6 μm. Since SiGe has already been introduced into CMOS production, the SiGe-based optical modulator presented here is highly CMOS-compatible. Thus, strained SiGe technology is easily applicable to most Si optical modulators based on the plasma dispersion effect and free-carrier absorption. We expect that the introduction of greater strain by increasing the Ge fraction in SiGe will enable the further enhancement of the plasma dispersion effect and free-carrier absorption. Although the modulation speed based on carrier-injection is relatively slower than those based on carrier-depletion and carrier-accumulation due to the slow minority carrier response time, we can expect a modulation speed above 10 Gbps by the pre-emphasis method^28^,^29^ even for the strained SiGe optical modulator. It is worth noting that the strain-induced enhancement of the free-carrier effects by strained SiGe is also applicable for accumulation or depletion type modulators to achieve further high speed modulation.

Hence, the strain-induced enhancement of the plasma dispersion effect and free-carrier absorption is one of the most promising technologies for boosting the performance of Si-based optical modulators.

## Methods

### Device simulation

The device structure of the strained SiGe-based optical modulator consists of a 115-nm-thick and 600-nm-wide waveguide mesa with boron doped at a constant doping level of 10^16^ cm^−3^. A 30-nm-thick Si_1*-x*_Ge*_x_* layer is embedded in the center of the waveguide mesa, which can be coherently grown on Si (001) when the Ge fraction is up to 0.3^22^. *p*^+^ and *n*^+^ regions with a doping level of 10^20^ cm^−3^ are formed on both sides of the waveguide mesa for carrier injection. The optical confinement factor of the SiGe layer is estimated to be 19% by finite-difference mode analysis. The carrier concentration is calculated by taking into account recombination processes such as Shockley-Read-Hall (SRH) and Auger recombination^30^,^31^,^32^ when electrons and holes are injected by applying a forward-bias voltage between the *p^+^* and *n^+^* regions. We assumed ohmic contacts in the *p^+^* and *n^+^* regions. After calculating the carrier concentration, the changes in the refractive index and absorption coefficient of SiGe and Si were calculated using the Drude model. Finally, the changes in the effective refractive index and absorption coefficient were obtained by calculating the overlap between the carrier distribution and the optical field distribution.

### Device fabrication and measurement

A 6-inch Si/SiGe/Si-on-insulator wafer was prepared by epitaxial growth on a commercially available (001) Si-on-insulator (SOI) wafer with a 2-μm-thick buried oxide (BOX) layer. First, a 260-nm-thick SOI layer was thinned to 100 nm by thermal oxidation. Then, a 30-nm-thick pseudomorphic Si_0.77_Ge_0.23_ layer and a 70-nm-thick Si layer were grown by chemical vapor deposition (CVD). The *pin* injection-type optical modulator was fabricated by the conventional Si CMOS process. First, a straight rib waveguide with a Si/SiGe/Si core was formed by deep ultraviolet (DUV) lithography and dry etching. Then, the ion implantation of boron and phosphorus was carried out to form the *p*^+^ and *n*^+^ regions for the fabrication of a *pin* junction, respectively, which was followed by activation annealing at 1000°C for 30 min in nitrogen atmosphere. Finally, the contact pads for the *p^+^* and *n^+^* regions were formed by the thermal evaporation of aluminum.

The amount of strain in the SiGe layer after device fabrication was evaluated by Raman spectroscopy. It is well known that biaxial strain is partially relaxed when a biaxially strained layer is etched into a narrow mesa with submicron width such as optical waveguides. Ge diffusion during the high-temperature activation annealing also reduces the strain in the SiGe layer. As a result, the Ge fraction and compressive strain in the SiGe layer after device fabrication are approximately 14% and 0.48%, respectively, corresponding to 85%-strained Si_0.86_Ge_0.14_ (Supplementary Fig. S5).

The optical attenuation was measured by injecting current to evaluate the enhancement of free-carrier absorption in the strained SiGe layer. Continuous-wave (CW) TE-polarized light in the wavelength range from 1.34 to 1.64 μm was coupled to the waveguide through a lensed fiber. Then, the output power was monitored using an InGaAs photodetector while changing the injection current. To calibrate the total coupling loss of approximately -30 dB, the insertion loss without injecting current was subtracted from the loss measured at the various injected currents.

## Author Contributions

Y.K. proposed the device design and process, designed the experiment, fabricated the samples, collected the data, and analyzed the data; T.O. and M.H. fabricated the epitaxial Si/SiGe on Si-on-insulator semiconductor substrates; M.T. and S.T. managed the research and supervised the experiment; all the authors discussed the results and commented on the manuscript.

## Supplementary Material

Supplementary Information Supplementary

## Figures and Tables

**Figure 1:**
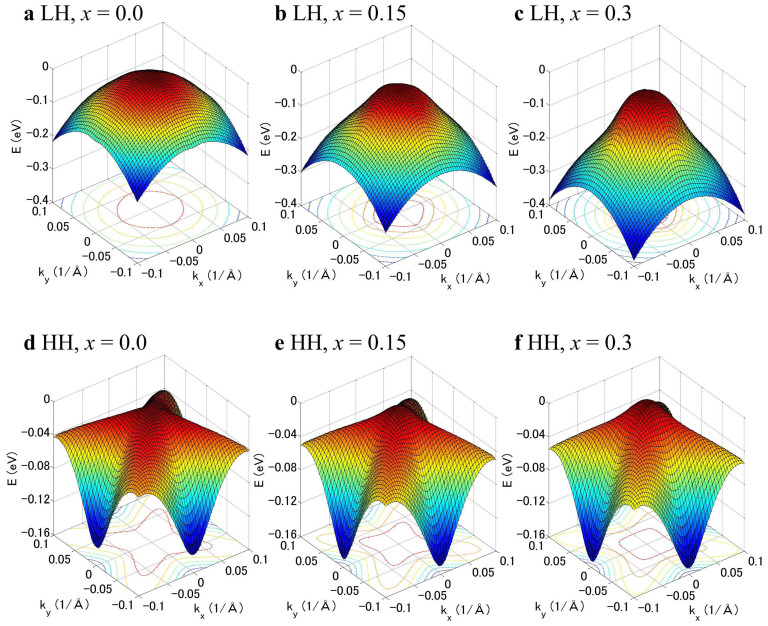
Valence band structures and constant-energy contours of Si_1-*x*_Ge*_x_* grown on Si (001) as a function of wavevector in the *k_x_* [100]-*k_y_* [010] plane calculated by the six-band *kp* method. The top three figures a, b, and c are LH band structures and the bottom three figures d, e, and f are HH band structures of Si_1-*x*_Ge*_x_* at *x* = 0 (Si), 0.15, and 0.3, respectively. The energy surfaces of the LH and HH bands become sharp at the minimum energy Γ point with increasing Ge fraction, indicating that biaxial compressive strain reduces the effective hole mass.

**Figure 2:**
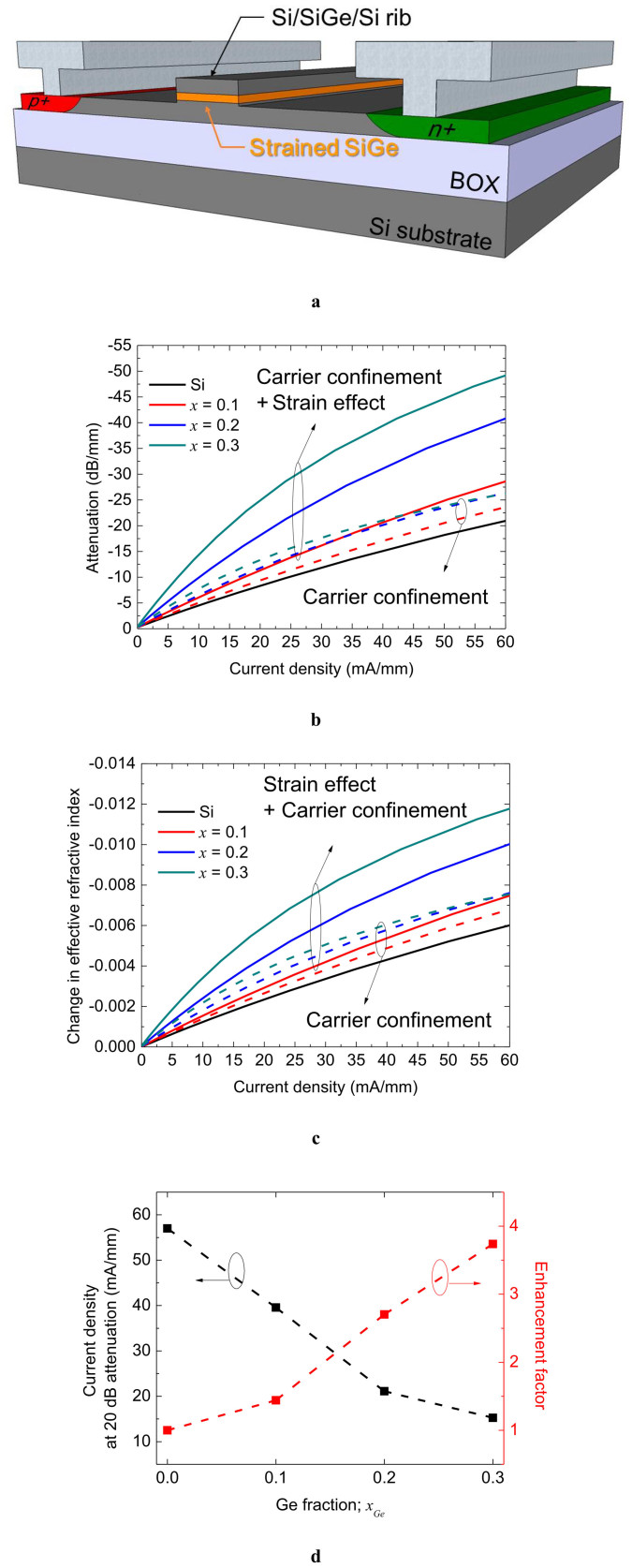
Simulation results of the *pin* injection-type strained SiGe optical modulator. (a), Device structure of the *pin* injection-type optical modulator with a Si/SiGe/Si heterostructure. (b), Attenuation characteristics as a function of injected current density to the in-line modulator with Ge fractions of 0.0 to 0.3. (c), Change in refractive index as a function of current density injected to in-line modulator with Ge fractions of 0.0 to 0.3. (d), Current density required for 20 dB attenuation (left-axis) and its enhancement factor (right axis) as a function of Ge fraction; the efficiency of the Si_0.7_Ge_0.3_-based in-line intensity modulator is predicted to be approximately 3.7 times as large as that of the Si-based modulator.

**Figure 3:**
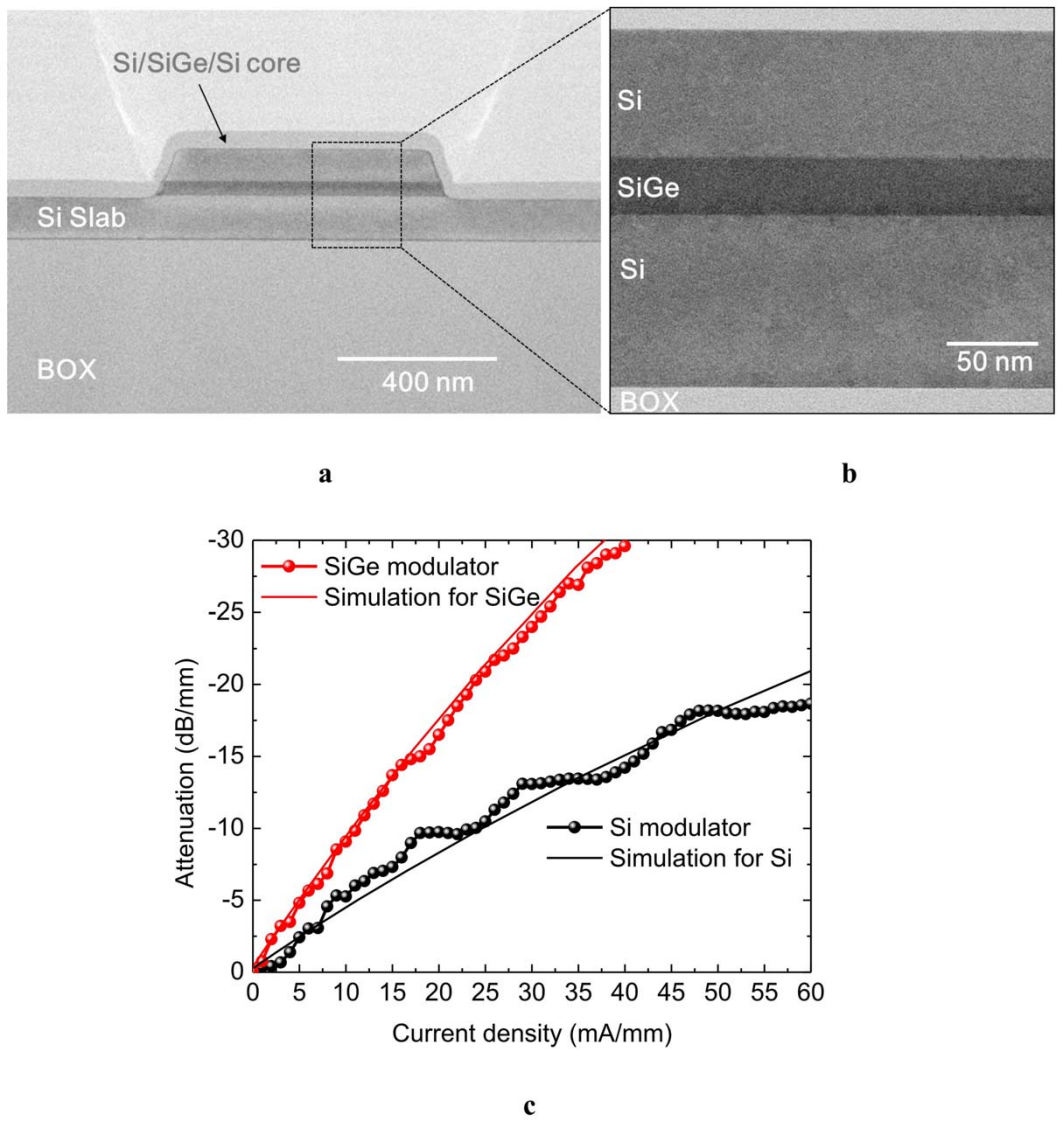
Experimental results of the *pin* injection-type strained SiGe optical modulator. (a), Cross-sectional TEM image of the Si/SiGe/Si waveguide, (b), TEM image of the Si/SiGe/Si heterostructure, and (c), Attenuation characteristics of the SiGe and Si modulators. Experimental results are shown by circles and simulated results are shown by lines. The modulation efficiency of the SiGe device is more than twice as large as that of the Si control device.

**Figure 4:**
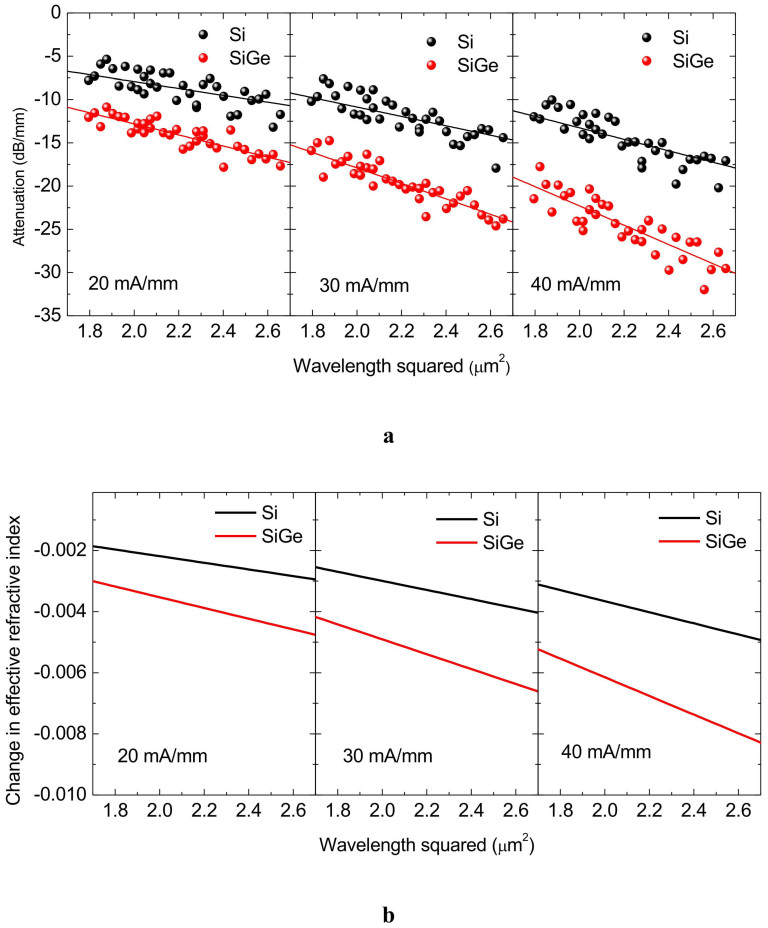
Wavelength dependence of modulation characteristics. (a), Measured attenuation of the SiGe and Si modulators with current densities of 20, 30, and 40 mA/mm. (b), Change in the effective refractive index calculated using the Kramers-Kronig relations from the measured wavelength dependence of the attenuation.

**Figure 5:**
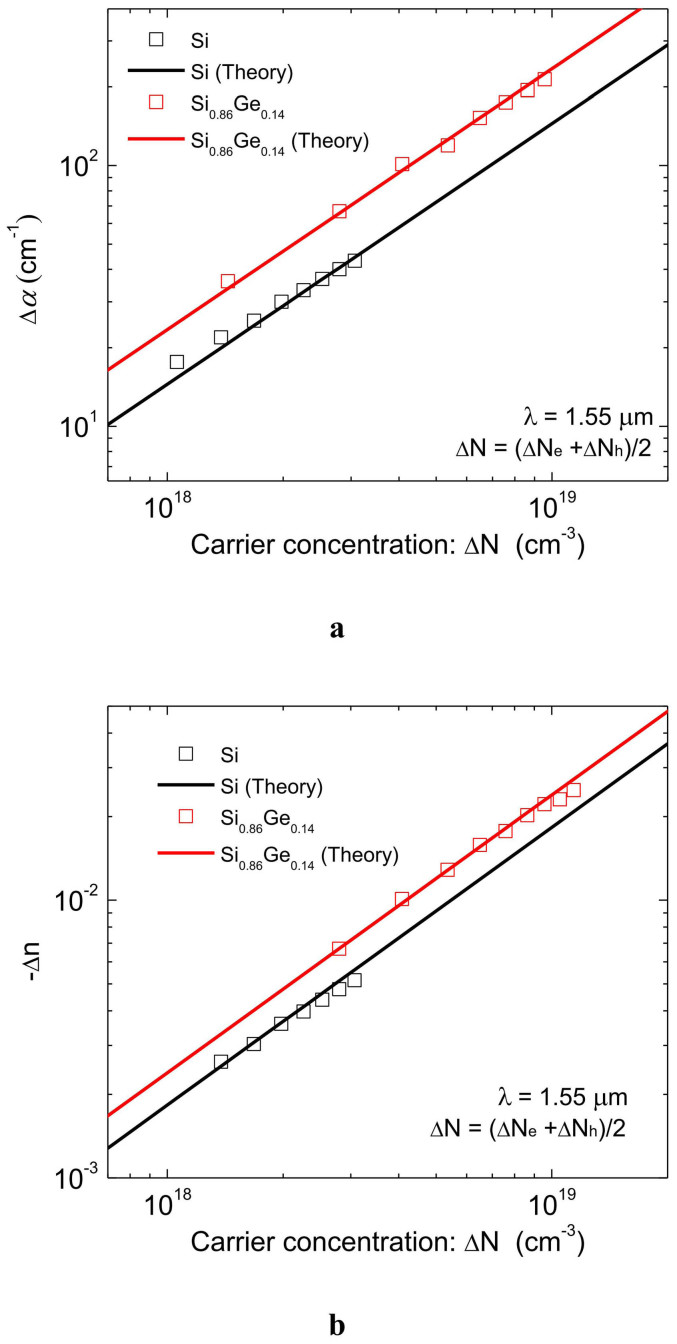
Changes in optical constants of Si_0.86_Ge_0.14_ and Si as functions of carrier concentration. (a), Change in absorption coefficient and (b), Change in refractive index. The experimental results and the theoretical values calculated using the Drude model are in good agreement, indicating that the plasma dispersion effect and free-carrier absorption are enhanced by strain-induced mass modulation in strained SiGe.
