# Supplementary material for: Strain-induced enhancement of plasma dispersion effect and free-carrier absorption in SiGe optical modulators
Source: Sci Rep. 2014 Apr 15;4:4683. doi: 10.1038/srep04683 (PMC3986731; doi:10.1038/srep04683)
Supplement: Supplementary Information — Supplementary [file srep04683-s1.doc]

**Supplementary information**

**Strain-induced enhancement of plasma dispersion effect and free-carrier absorption in SiGe optical modulators**

Younghyun Kim^1*^, Mitsuru Takenaka^1^, Takenori Osada^2^, Masahiko Hata^2^, and Shinichi Takagi^1^

^1^ *Department of Electrical Engineering and Information Systems, The University of Tokyo, 7-3-1 Hongo, Bunkyo-ku, Tokyo 113-8656, Japan*

^2^ *Sumitomo Chemical Co., Ltd., 6 Kitahara, Tsukuba, Ibaraki 300-3294, Japan*

^*^ *E-mail: yhkim@mosfet.t.u-tokyo.ac.jp*

**Contents**

- Calculation of conductivity effective mass of holes in compressively strained SiGe using *k⋅p* method
- Estimation of enhancement factor of changes in refractive index and absorption coefficient
- Fundamental TE mode in Si/SiGe/Si core
- Carrier confinement in strained SiGe
- Analysis of strain relaxation of SiGe layer
- Extraction of changes in refractive index from absorption coefficient spectrum by the Kramers-Kronig relations

**Calculation of conductivity effective mass of holes in compressively strained SiGe using *k⋅p* method**

To calculate the conductivity effective mass of holes in compressively strained SiGe, the valence band structure of SiGe coherently grown on (001) Si is calculated by the *k⋅p* method. The following 6 × 6 Hamiltonian is used to evaluate strain-induced coupling between the heavy-hole (HH) bands, light-hole (LH) bands, and spin-orbit split-off (SO) bands^1^:

$H\left(\text{k}\right)=\left[\begin{matrix} P+Q & -S & R & 0 & -\frac{1}{\sqrt{2}}S & \sqrt{2}R \\ -S^{\dagger} & P-Q & 0 & R & -\sqrt{2}R & \sqrt{\frac{3}{2}}S \\ R^{\dagger} & 0 & P-Q & S & \sqrt{\frac{3}{2}}S^{\dagger} & \sqrt{2}Q \\ 0 & R^{\dagger} & S^{\dagger} & P+Q & -\sqrt{2}R^{\dagger} & -\frac{1}{\sqrt{2}}S^{\dagger} \\ -\frac{1}{\sqrt{2}}S^{\dagger} & -\sqrt{2}Q & \sqrt{\frac{3}{2}}S & -\sqrt{2}R & P+\Delta& 0 \\ \sqrt{2}R^{\dagger} & \sqrt{\frac{3}{2}}S^{\dagger} & \sqrt{2}Q & -\frac{1}{\sqrt{2}}S & 0 & P+\Delta\end{matrix}\right]$ (1)

with *P = P_k_ + P_ε_*, *Q = Q _k_ + Q_ε_*, *R = R_k_ + R_ε_*, *S = S_k_ + S_ε_*, and

, ,

, ,

$P_{\varepsilon}= -a_{V}Tr\left(\varepsilon\right)$, $Q_{\varepsilon}= -\frac{b}{2}\left(\varepsilon_{xx}+\varepsilon_{yy}-2\varepsilon_{zz}\right)$, $R_{\varepsilon}= \frac{\sqrt{3}}{2}b\left(\varepsilon_{xx}-\varepsilon_{yy}\right)-id\varepsilon_{xy}$,

$S_{\varepsilon}= -d\left(\varepsilon_{xx}-\varepsilon_{yz}\right)$,

where Δ is the spin-orbit splitting, *γ*_1_, *γ*_2_ , and *γ*_3_ are the Luttinger parameters, and *b* and *d* are the Bir-Pikus shear deformation potentials. Table S1 shows the material parameters of Si and Ge employed in this study. All values are from references^2,3^, except for the valence band parameters^4^. The unit of the valence band parameters is *ħ*/2*m_0_*, where *ħ* is the reduced Planck constant and *m_0_* is the electron mass. The values for SiGe are obtained by linear interpolation of those for Si and Ge, with the exception of the lattice constant of Si_1_*_−x_*Ge*_x_*, which is approximately expressed as

*a_SiGe_*(*x*) = *a_Si_* + 0.200326·*x* (1 − *x*) + ( *a_Ge_* − *a_Si_*) *x* ^2^ , (2)

where *x* is the Ge mole fraction^5^. The three band-edge levels HH, LH, and SO are obtained by solving det[*H*(***k***) – *E*] = 0 and then, the in-plane conductivity effective mass of holes in SiGe is calculated.

Table S1. Material parameters of Si and Ge

| Symbol | Quantity | Units | Si | Ge |
| --- | --- | --- | --- | --- |
| *a* | Lattice constant | Å | 5.43 | 5.65 |
| *C_11_* | Elastic constants | Mbar | 1.658 | 1.285 |
| *C_12_* |  | Mbar | 0.539 | 0.483 |
| *a_v_* | Hydrostatic deformation potential | eV | 2.46 | 1.24 |
| *b* | Bir-Pikus shear deformation potential | eV | -2.2 | -2.3 |
| *d* |  | eV | -5.1 | -5.0 |
| *Δ* | Spin-orbit split-off energy | eV | 0.044 | 0.295 |
| *L* | Valence band parameters | *ћ*/2*m*_0_ | -5.53 | -30.53 |
| *M* |  |  | -3.64 | -4.64 |
| *N* |  |  | -8.32 | -33.64 |

The conductivity effective masses of holes and electrons as a function of the Ge fraction are shown in Fig. S1. From the calculated valance band energies of LH and HH, the conductivity effective hole masses of strained and relaxed SiGe are obtained. The conductivity effective mass of electrons is taken from reference^6^. Compared with the mass of Si (*x* = 0), the conductivity hole mass of relaxed SiGe deceases with increasing Ge fraction. Moreover, the conductivity hole mass rapidly decreases owing to the compressive strain. On the other hand, the in-plane mass of electrons in Δ valleys remains almost constant regardless of the Ge fraction in the case of applying biaxial compressive strain.

**Figure S1. Calculated in-plane conductivity effective hole masses of compressively strained Si_1_*_-x_*Ge*_x_* grown on Si (black) and relaxed Si_1_*_-x_*Ge*_x_* (red), and electron masses for strained SiGe^6^ (blue).** Comparing the hole masses of relaxed and strained SiGe, the compressive strain has a large effect on reducing the conductivity hole mass.

**Estimation of enhancement factor of changes in refractive index and absorption coefficient**

The plasma dispersion effect and free-carrier absorption are expressed by the Drude model, which shows that changes in the refractive index and absorption coefficient arise from a change in the plasma frequency of free carriers, which is dependent on the number of free carriers and their conductivity effective masses. Thus, the changes in the refractive index (Δ*n*) and absorption coefficient (Δ*α*) are expressed as

Δ*n* = －(*e*^2^*λ*^2^/8*π*^2^*c*^2^*ε*_0_*n*)[ Δ*N_e_*/*m^*^_ce_* +Δ*N_h_*/*m^*^_ch_*] (3)

Δ*α* = (*e*^3^*λ*^2^/4*π*^2^*c*^3^*ε*_0_*n*)[ Δ*N_e_*/*m^*^_ce_*^2^*μ_e_* +Δ*N_h_*/*m^*^_ce_*^2^*μ_h_*], (4)

where *e* is the elementary charge, *ε*_0_ is the permittivity in vacuum, *c* is the speed of light in vacuum, *λ* is the wavelength, *n* is the unperturbed refractive index, *m^*^_ce_* and *m^*^_ch_* are the conductivity effective masses of electrons and holes, and *μ_e_* and *μ_h_* are the mobilities of electrons and holes, respectively^7^.

According to the Drude model, the changes in the optical constants are inversely proportional to the conductivity effective mass for Δ*n* and the square of the conductivity effective mass multiplied by the mobility for Δ*α*. Therefore, the changes in the optical constants are expected to be enhanced by reducing the conductivity effective masses.

Figures S2(a) and S2(b) show the enhancement of changes in the refractive index and absorption coefficient in compressively strained SiGe, respectively in which the mobilities of electrons^4^ and for holes^8^were taken from the literatures. The refractive index of SiGe was taken from the reference^9^. The changes in the optical constants caused by holes increase with an increase in the Ge fraction because the conductivity hole mass of SiGe is reduced by compressive strain as shown in Fig. S1. On the other hand, there is no enhancement of the change in the refractive index caused by electrons because there is almost no modulation of the electron mass by strain, as shown in Fig. S1. The reduction in the electron mobility with increasing Ge fraction slightly contributes to the enhancement of the change in the absorption coefficient by electrons, but this contribution is much smaller than that of holes. When we consider a SiGe optical modulator into which carriers are injected through a forward-biased *pin* junction, the same amounts of electrons and holes exist in the SiGe layer owing to the charge neutrality condition. Thus, the average changes in the optical constants caused by injected electrons and holes in the *pin* structure are reduced, as shown in Fig. S2. However, the average enhancement factors of Δ*n* and Δ*α* still increase with increasing Ge fraction and are expected to be approximately 1.6 for Δ*n* and 2.1 for Δ*α* in Si_0.7_Ge_0.3_.

(a) (b)

**Figure S2.** Enhancement factors for changes in optical constants. (a) Refractive index and (b) Absorption coefficient as functions of the Ge fraction. The changes in the optical constants increase with increasing Ge fraction, and Δ*n* and Δ*α* are expected to be enhanced by as much as 1.6 and 2.1, respectively, in compressively strained Si_0.7_Ge_0.3_.

**Fundamental TE mode in Si/SiGe/Si core**

Figure S3 shows the electric field distribution of fundamental transverse electric (TE) mode calculated by the finite-difference method. The optical confinement factor of the SiGe layer is estimated to be 19% by finite-difference mode analysis.


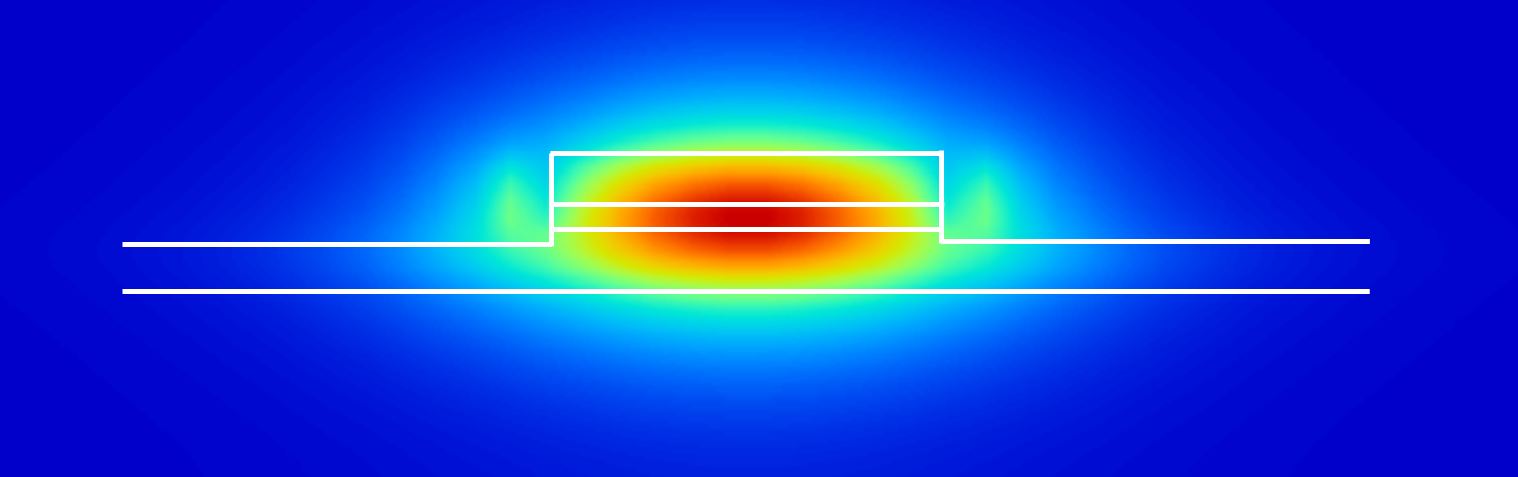


**Figure S3.** Electric field distribution of the fundamental TE mode of the SiGe optical modulator. The SiGe layer is placed at the center of the rib to obtain the maximum optical confinement factor.

**Carrier confinement in strained SiGe**

We show the carrier concentrations calculated by TCAD simulation in Fig. S4(a) as a function of the current density injected into the Si/SiGe/Si heterostructure modulator. It is found that the carrier concentration in the SiGe layer is much larger than that in the Si layer because the injected carriers are confined in the SiGe layer owing to the band offset between Si and SiGe, as shown in Fig. S4(b), which also contributes to improving the modulation efficiency of Si-based optical modulators^10^. The carrier concentration in the SiGe layer increases by an order of magnitude as the Ge fraction increases from 0 to 0.3 because the band offset between Si and SiGe increases. Thus, it is possible to synergistically enhance the changes in the effective refractive index and absorption coefficient by accumulating the injected carriers in the SiGe layer, where the plasma dispersion effect and free-carrier absorption are larger than those of Si.

(a)


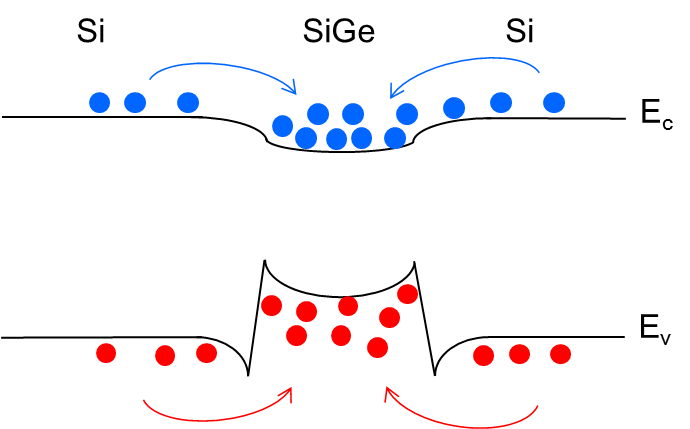


(b)

**Figure S4.** Carrier confinement in SiGe layer. (a) Carrier concentrations at Si and SiGe layers as a function of injected current density with the Ge fractions of 0.0 to 0.3. (b) Bandstructure of Si/SiGe/Si heterostructure illustrating carrier confinement in the SiGe layer.

**Analysis of strain relaxation of SiGe layer**

The amount of strain in the SiGe layer in the fabricated device was evaluated by Raman spectroscopy. It is well known that the biaxial strain is partially relaxed when a biaxially strained layer is etched into a narrow mesa with submicron width such as optical waveguides. Ge diffusion during high-temperature activation annealing also causes strain relaxation in the SiGe layer. Figure S5 shows the amount of strain in SiGe measured before and after dopant activation annealing at 1000 ºC for 30 min. The effect of the waveguide width on strain is also shown in Fig. S5 for mesa widths from 600 nm to 1900 nm. The dotted line in Fig. S5 shows the ideal strain curve of fully strained SiGe. The wide-mesa SiGe exhibits full strain before annealing, while the amount of strain decreases after annealing because the Ge fraction is reduced by Ge diffusion into the Si layers. The cross-sectional TEM image of the Si/SiGe/Si heterostructure in the inset of Fig. S5 indicates that the thickness of the SiGe layer after annealing is increased to approximately 50 nm. The dry etching of the 600-nm-wide waveguide also causes partial strain relaxation by 15%, as shown in Fig. S5. Thus, the Ge fraction and compressive strain of the SiGe layer in the fabricated device are approximately 14% and 0.48%, respectively, corresponding to 85%-strained Si_0.86_Ge_0.14_.

**Figure S5.** Compressive strain as a function of Ge fraction for different waveguide widths and cross-sectional TEM image of Si/SiGe/Si heterostructure after annealing process (inset).

**Extraction of changes in refractive index from absorption coefficient spectrum using Kramers-Kronig relations**

The Kramers-Kronig relations are the well-known physical relationship between the change in refractive index and absorption coefficient, and isbeen described as follows:

, (5)

where *c* is the speed of light, *ω* is the angular frequency, and *P* is the principal value of the integral. Here, the change in absorption coefficient change is defined as follows:

$\Delta\alpha\left(\omega, \Delta N\right) = \Delta\alpha\left(\omega, \Delta N\right) - \Delta\alpha\left(\omega, 0\right)$, (6)

where Δ*N* is the change in carrier concentration. Hence, equation (5) can be used to extract the change in the refractive index from the spectrum showing the change in the absorption coefficient. Figures S6(a) and (b) show the change in the effective refractive index of the Si and SiGe optical modulators, calculated from the experimentally obtained the changes in the absorption coefficient spectra, respectively. As described in equation (3), the calculated the changes in the effective refractive index have linear relationship with the square of the wavelength. It is clearly observed in Fig. S6 that the changes for the SiGe device have larger slopes than those for the Si device at all current densities, indicating that the plasma dispersion effect of strained SiGe is larger than that of Si.

(a) (b)

**Figure S6.** Changes in effective refractive index of (a) Si and (b) SiGe optical modulators calculated using the Kramers-Kronig relations from the measured spectra of the change in absorption coefficient with injected current density for current densities of 5 to 50 mA/mm in 5 mA steps.

**References**

1. Chao, C. Y. & Chuang, S. L. Spin-orbit-coupling effects on the valence-band structure of strained semiconductor quantum wells. *Phys. Rev. B* **46**, 4110–4122 (1992).
2. Yu, P. Y. and Cardona, M. *Fundamentals of Semiconductors*. (New York: Springer-Verlag, 1995).
3. Shiraki, Y. & Usami, N. *Silicon-Germanium (SiGe) Nanostructures*. (Cambridge, U.K.: Woodhead Publishing, 2011).
4. Fischetti, M. V. & Laux, S. E. Band structure, deformation potentials, and carrier mobility in strained Si, Ge, and SiGe alloys. *J. Appl. Phys.* **80**, 2234–2252 (1996).
5. Dismukes, J. P., Ekstrom, L. & Paff, R. J. Lattice parameter and density in germanium silicon alloys, *J. Phys. Chem.* **68**, 3021–3027 (1964).
6. Martin, M. R. & Vogl, P. Electronic-band parameters in strained Si*_1-x_*Ge*_x_* alloys on Si*_1-y_*Ge*_y_* substrates. *Phys. Rev. B* **48**, 276–287 (1992).
7. Soref, R. A. & Bennett, B. R. Electrooptical effects in silicon. *IEEE J. Quantum Electron.* **23**, 123–129 (1987).
8. Arora, N. D., Hauser, J. R. & Roulston, D. J. Electron and hole mobilities in silicon as a function of concentration and temperature. *IEEE Trans. Electron Devices* **29**, 292–295 (1982).
9. Schaffler, F. *Properties of Advanced Semiconductor Materials GaN, AlN, InN, BN, SiC, SiGe*. (New York: Wiley, 2001).
10. Sekiguchi, S., Kurahashi, T., Zhu, L., Kawaguchi, K. & Morito, K. Compact and low power operation optical switch using silicon-germanium/silicon hetero-structure waveguide. *Opt. Express* **20**, 17212–17219 (2012).
